# D-dimer in the diagnosis of periprosthetic joint infection: a systematic review and meta-analysis

**DOI:** 10.1186/s13018-020-01761-z

**Published:** 2020-07-16

**Authors:** Guangxu Lu, Tong Li, Haoqi Ye, Shujin Liu, Peng Zhang, Wenliang Wang

**Affiliations:** 1 Department of Logistics University of PAP, Tianjin, 300309 China; 2 Department of Orthopedics, Characteristic Medical Center of PAP, Tianjin, 300162 China; 3 Department of Orthopedics, Er Quan Hospital of PAP, Wuxi, 214000 China

**Keywords:** Periprosthetic joint infection, D-dimer, Arthroplasty, Diagnosis, Meta-analysis

## Abstract

**Background:**

D-dimer, a coagulation-related indicator, has recently been used as a tool for the diagnosis of periprosthetic joint infection (PJI), but its reliability is uncertain. The purpose of this systematic review and meta-analysis was to explore the accuracy of D-dimer in the diagnosis of PJI after joint arthroplasty.

**Methods:**

We systematically searched the MEDLINE, EMBASE, and Cochrane databases for relevant literature about D-dimer in the diagnosis of PJI. QUADAS-2 was used to assess the risk of bias and clinical applicability of each included study. We used the bivariate meta-analysis framework to pool the sensitivity, specificity, positive likelihood ratio (PLR), negative likelihood ratio (NLR), diagnostic odds ratio (DOR), and area under the SROC curve (AUC). Univariate meta-regression and subgroup analyses were performed to explore the sources of heterogeneity.

**Results:**

We included 8 eligible studies. The pooled diagnostic sensitivity and specificity were 0.82 (95% CI, 0.70–0.89) and 0.70 (95% CI, 0.55–0.82), respectively. The pooled PLR, NLR, and DOR were 2.7 (95% CI, 1.7–4.4), 0.26 (95% CI, 0.15–0.46), and 10 (95% CI, 4–25), respectively. The AUC was 0.83 (95% CI, 0.8–0.86). Serum D-dimer might have higher diagnostic accuracy than plasma D-dimer for PJI (pooled sensitivity: 0.88 vs 0.67; pooled specificity: 0.76 vs 0.61).

**Conclusions:**

D-dimer has limited performance for the diagnosis of PJI.

## Introduction

Periprosthetic joint infection (PJI) is a rare and devastating complication that affects 0.7–2.4% of patients after hip or knee arthroplasty [1–3]. PJI not only affects the quality of life of infected patients but also increases the risk of death [4].

Because the typical clinical manifestations of patients with PJI may not appear and pain can be caused by other diseases, PJI is difficult to diagnose. The Musculoskeletal Infection Society (MSIS) formulated diagnostic criteria for PJI and tried to reduce the incidence rate of this dreaded complication [5, 6]. In 2018, the International Consensus Meeting (ICM) modified the criteria and added D-dimer and alpha-defensin into the new definition of PJI for the knee and hip joint [7] (Table 1).

**Table 1:** MSIS criteria for diagnosis of PJI (modified by ICM in 2018) [7]

**Major criteria**	1. Two positive periprosthetic cultures with phenotypically identical organisms			
	2. A sinus tract communicating with the joint			
**Minor criteria**	3. Preoperative diagnosis		Score	Decision
	Serum	1).CRP (> 1 mg/dL) OR D-dimer (> 850 ng/mL)	2	≥ 6: Infected 2–5: Possibly infected 0–1: Not infected
		2). ESR (> 30 mm/h)	1	
	Synovial	1). Synovial WBC count (> 3000 cells/uL) or LE +	3	
		2). Alpha-defensin (signal-to cut-off ratio > 1)	3	
		3). Synovial PMN (%) (> 80%)	2	
		4). Synovial CRP (> 6.9 mg/L)	1	
	4. Intraoperative diagnosis.		Score	≥ 6: Infected 4–5: Inconclusive ≤ 3: Not infected
	1). Preoperative score		–	
	2). Histology		3	
	3). Purulence		3	
	4). Single culture		2	

*PJI* is present when 1 of the major criteria is met

*CRP* C-reactive protein, *ESR* erythrocyte sedimentation rate, *WBC* white blood cell, and *PMN%* polymorphonuclear neutrophil percentage

D-dimer is a specific degradation product of fibrin monomer that is crosslinked by activating factor XIII and then hydrolyzed by fibrinolytic enzyme [8]. It is a specific marker of the fibrinolysis process and mainly reflects the function of fibrinolysis [8]. A study suggested that D-dimer could be used to determine prognosis in systemic sepsis [9]. D-dimer levels continue to rise due to the host’s inflammatory response to infection in sepsis [9].

Currently, some studies have examined the diagnostic value of D-dimer for PJI, but diagnostic accuracy varies in different studies. Therefore, the purpose of this systematic review and meta-analysis was to evaluate the diagnostic accuracy of D-dimer for PJI.

## Materials and methods

**Fig. 1:**
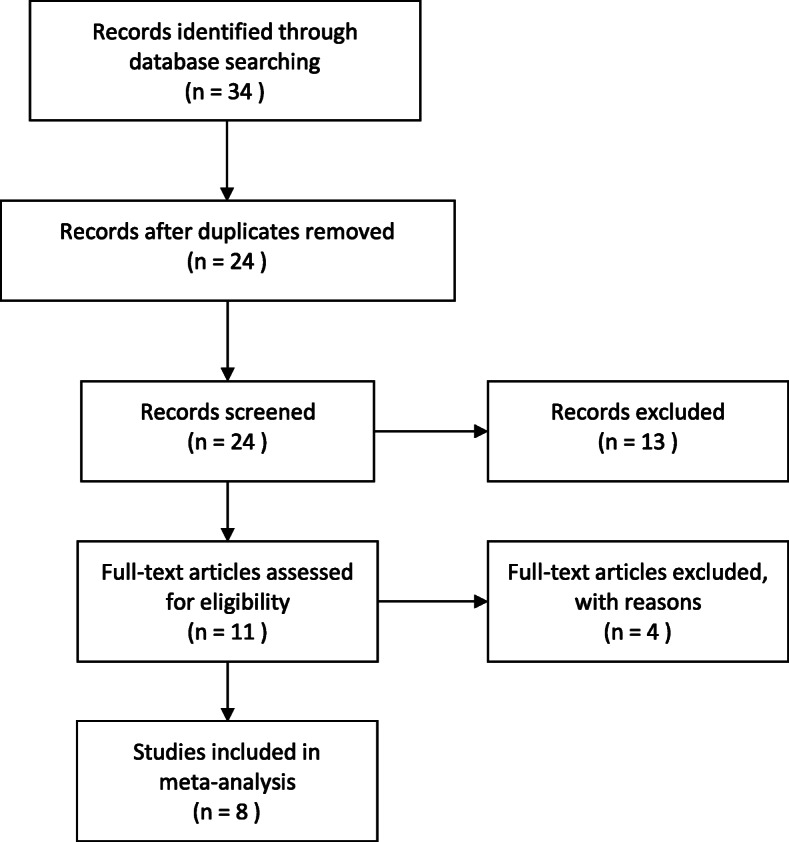
PRISMA flow diagram

This systematic review and meta-analysis strictly followed the Preferred Reporting Items for Systematic Reviews and Meta-Analyses (PRISMA) guidelines [10] (Fig. 1).

### Search strategy

We systematically searched all literature about D-dimer in the diagnosis of PJI in MEDLINE, Embase, and the Cochrane Library (from the inception of each database until November 2019), without language restrictions. The search strategies are shown in Table 2.

**Table 2:** Search strategy on MEDLINE, Embase, and Cochrane

**PubMed and Cochrane**	
#1 ((((((((((((joint arthroplasty) OR joint replacement) OR knee replacement) OR hip replacement) OR hip arthroplasty) OR knee arthroplasty) OR Arthroplasty, Replacement, Knee) OR Arthroplasty, Replacement) OR Arthroplasty, Replacement, Shoulder) OR Arthroplasty, Replacement, Hip) OR shoulder replacement) OR shoulder arthroplasty #2 ((periprosthetic infection) OR prosthetic joint infection) OR periprosthetic joint infection #3 ((diagnostic test) OR test) OR diagnosis #4 (((((((fibrin fragment D) or D-dimer fibrin) or D-dimer fragments) or fibrin fragment D1 dimer) or fibrin fragment DD) or D-dimer) or fibrin fragment D-dimer) #1 and #2 and #3 and #4	
**EMBASE**	
#1 ‘joint arthroplasty' OR 'joint replacement' OR 'knee replacement' OR 'hip replacement' OR 'hip arthroplasty' OR 'knee arthroplasty' OR 'replacement arthroplasty' OR 'shoulder replacement' OR 'shoulder arthroplasty' #2 ‘diagnostic test' OR 'diagnosis' OR test #3 ‘periprosthetic infection' OR 'prosthetic joint infection' OR 'periprosthetic joint infection' #4 ‘fibrin fragment d'/expr OR 'fibrin fragment d' OR 'd-dimer fibrin' OR 'd-dimer fragments' OR 'fibrin fragment d1 dimer' OR 'fibrin fragment dd' OR 'd dimer'/exp OR 'd dimer' OR 'fibrin fragment d-dimer' #1 and #2 and #3 and #4	

### Eligibility criteria

We included all studies that reported the accuracy of D-dimer in the diagnosis of PJI after hip or knee arthroplasty and used the MISS or modified MISS criteria. Studies lacking sensitivity and specificity values and those that had duplicated data were excluded.

Two authors independently scanned the titles, abstracts, and full texts sequentially and screened the literature based on the eligibility criteria. The third author settled any disagreements that arose.

### Data extraction

Two authors independently classified all studies and extracted data using standardized scales. We extracted all baseline data (author name, publication year, country, average age, sex distribution, BMI, joint type, patient exclusion criteria, diagnostic criteria, etc.) and outcome indicators (sensitivity, specificity, PLR, NLR, DOR, AUC, etc.). The third author resolved any disagreements that arose.

### Quality evaluation

The quality of each included study was evaluated using the QUADAS-2 tool [11], which mainly includes four parts: patient selection, indicator testing, reference standard, and flow and timing. The first three parts are also needed to evaluate clinical practicability. According to the answers (“yes,” “no,” or “uncertain”) to the relevant landmark questions included in each part, the risk of bias level was determined as “low,” “high,” or “uncertain.” Two authors independently evaluated the quality, and the third author decided the final result in the event of any divergences.

### Statistical analysis

We used the bivariate meta-analysis framework to pool the sensitivity, specificity, PLR, NLR, DOR, and AUC by using the “Midas” command [12]. Compared with the traditional summary ROC curve, the bivariate model is a development and expansion [13]. The joint modeling of sensitivity and specificity is used as the starting point for the analysis, and a random effects model is used [13]. Thus, the diagnostic accuracy may be more reliable with this method [14]. The *I*^2^ statistic was used to estimate the heterogeneity among studies. The value of *I*^2^ is between 0 and 100%. An *I*^2^ value of < 50% indicates low heterogeneity, while an *I*^2^ value of > 50% indicates high heterogeneity.

When there was high heterogeneity, we evaluated the threshold effect through the Spearman correlation coefficient of the logarithm of sensitivity and 1-specificity. When the *P* value was < 0.05, the threshold effect was considered significant. At the same time, we used univariate meta-regression to find the potential sources of heterogeneity. Then, we conducted a subgroup analysis to further investigate the source of heterogeneity. A test for publication bias (Deeks’ funnel plot) was also used to analyze the sources of heterogeneity. When the *P* value was < 0.05, the tests for publication bias were considered statistically significant [15].

Stata 14.0 software and Meta-DiSc 1.4 were used for data analysis.

## Result

After a systematic search in the above databases, 34 studies were initially selected, and finally, 8 studies [16–23] were included according to the inclusion and exclusion criteria (Table 3). The 8 included studies were conducted in 2 countries (China and the USA) and included 1587 patients, involving 514 knee joints, 822 hip joints, and 50 extra-articular infections. A total of 457 patients were diagnosed with PJI, and the rate ranged from 17 to 45%. The average age of all the patients in the studies ranged from 61.5 to 68.9 years, with 33–53% males. All 8 studies were published in the last 3 years, and there was no patient overlap in these studies.

**Table 3:** Characteristics of included studies

Study	Year	Country	Study design	No. of patients^a^	Mean age	Mal/female	Site of arthroplasty	Exclusion criteria	Reference standard	Cut-off	Sample
Shahi et al. [20]	2017	USA	Prospective	57/245^b^	61.5	129/116	Knee (98) and hip (97)	A	MSIS	850 ng/ml	Serum
Fu et al. [22]	2019	China	Prospective	15/45	65.8	12/33	Knee (40) and hip (5)	B	MSIS	850 ng/ml	Plasma
Li et al. [19]	2019	China	Retrospective	95/565	61.7	248/317	Knee (153) and hip (412)	A	ICM	1250 ng/ml	Plasma
Xu et al. [18]	2019	China	Retrospective	129/318	NA	NA	Knee (63) and hip (23)	A	MSIS	1020 ng/ml	Plasma
Huang et al. [21]	2019	China	Retrospective	31/101	66.4	NA	Hip (101)	B	MSIS	850 ng/ml	Serum
Qin et al. [16]	2020	China	Prospective	55/122	65.2	53/69	Knee (44) and hip (78)	B	MSIS	1170 ng/ml	Serum
Xiong et al. [17]	2019	China	Prospective	26/80	62.3	32/48	Knee (47) and hip (33)	B	MSIS	760 ng/ml	Serum
Pannu et al. [23]	2020	USA	Retrospective	49/111	68.9	49/111	Knee (69) and hip (42)	A	ICM	850 ng/ml	serum

*A* does not exclude patients with rheumatoid arthritis, autoimmune diseases, tumors, smoking and obesity; *B* exclude patients with rheumatoid arthritis, autoimmune diseases, tumors, smoking, and obesity

*NA* not available

^a^The values are given as the number of patients with an infection/total number of patients in study

^b^50 of 245 patients were extra-articular infection

Four studies [16, 17, 20, 22] were prospective studies, and the other 4 studies [18, 19, 21, 23] were retrospective studies. In terms of the diagnostic threshold, 4 studies [20–23] used 850 퓊g/L, which was recommended by the ICM (2018) as the diagnostic threshold of D-dimer. Pannu et al. [23] also used 2300 ng/ml as the cut-off in their study. The remaining 4 studies [16–19] used 1250 ng/L, 1020 ng/L, 1170 ng/ml, and 760 ng/ml as the diagnostic thresholds. Four studies [21–23] determined the diagnostic threshold in advance, and the remaining studies [16–20] obtained the diagnostic threshold from the ROC curve. Three studies [18, 19, 22, 23], all from China, used plasma samples for the quantification of D-dimer, and 5 studies [16, 17, 20, 21, 23] used serum samples. Four studies [16, 17, 21, 22] excluded patients with rheumatoid arthritis, autoimmune diseases, tumors, smoker status, or obesity and the remaining 4 studies [18–20, 23] did not.

### Quality assessment

**Fig. 2:**
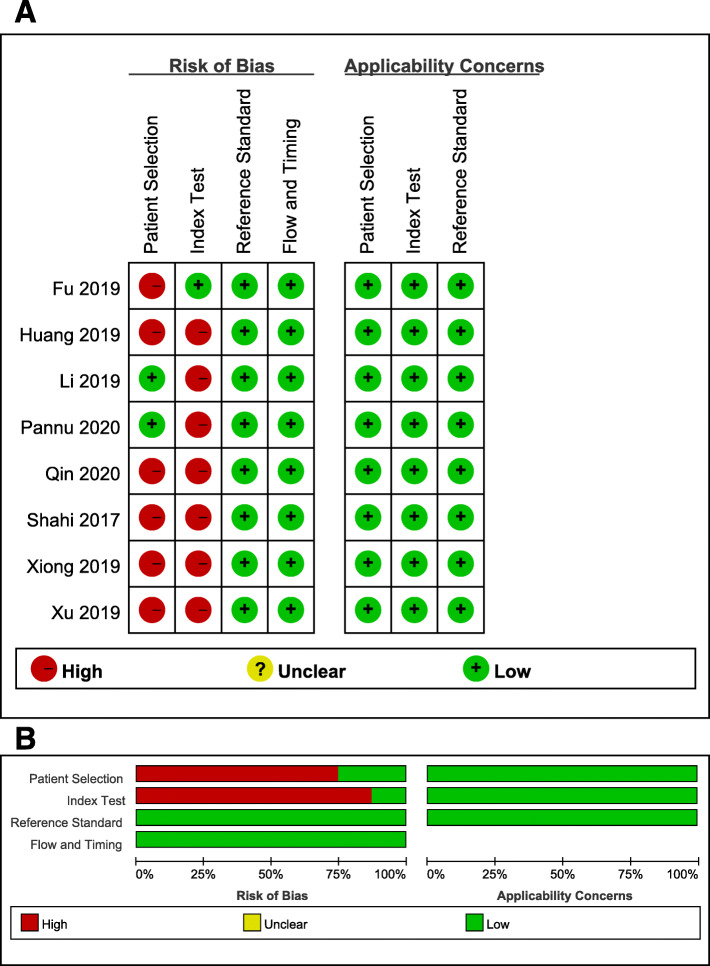
Risk of bias and applicability concerns summary. **a** Risk of bias and applicability concerns graph (**b**)

According to the QUADAS-2 tool, we evaluated the quality of all included studies (Table 4 and Fig. 2). The risk of bias in reference standards and flow and timing was low in all studies. Six studies [16–18, 20–22] were at high risk of bias for patient selection because of inappropriate discharge standards and case-control trials. Because retrospective studies and thresholds were not set in advance in 7 studies [16–21, 23], the bias of the index test was high. All studies scored between 6 and 9 (the total score is 10 points).

**Table 4:** QUADAS-2 evaluation

Study	QUADAS Score^*^																
	1	2	3	Bias	Appl.	4	5	Bias	Appl.	6	7	Bias	Appl.	8	9	10	Bias
Shahi et al. [20]	NC	0	1	High	Low	1	0	High	Low	1	1	Low	Low	1	1	1	Low
Fu et al. [22]	NC	0	0	High	Low	1	1	Low	Low	1	1	Low	Low	1	1	1	Low
Li et al. [19]	NC	1	1	Low	Low	0	0	High	Low	1	1	Low	Low	1	1	1	Low
Xu et al. [18]	NC	0	1	High	Low	0	0	High	Low	1	1	Low	Low	1	1	1	Low
Huang et al. [21]	NC	0	0	High	Low	0	1	High	Low	1	1	Low	Low	1	1	1	Low
Qin et al. [16]	NC	1	0	High	Low	1	0	High	Low	1	1	Low	Low	1	1	1	Low
Xiong et al. [17]	NC	1	0	High	Low	1	0	High	Low	1	1	Low	Low	1	1	1	Low
Pannu et al. [23]	1	1	1	Low	Low	0	1	High	Low	1	1	Low	Low	1	1	1	Low

The numbers in the top row correspond to the following questions: Domain 1: Patient selection. Numbers correspond with the following questions: (1) Was a consecutive or random sample of patients enrolled? (2) Was a case-control design avoided? (3) Did the study avoid inappropriate exclusions? Domain 2: Index test. Numbers correspond with the following questions: (4) Were the index test results interpreted without knowledge of the results of the reference standard? (5) If a threshold was used, was it pre-specified? Domain 3: Reference test. Numbers correspond with the following questions: (6) Is the reference standard likely to correctly classify the target condition? (7) Were the reference standard results interpreted without knowledge of the results of the index test? Domain 4: Flow and timing. Numbers correspond with the following questions: (8) Was there an appropriate interval between index test(s) and reference standard? (9) Did all patients receive a reference standard? (10) Did patients receive the same reference standard? (11) Were all patients included in the analysis?

*Number 1 indicates “yes,” and 0 indicates “no”; Bias risk: of bias; Appl.: concerns regarding applicability; NC: not clear

### Diagnostic value

**Fig. 3:**
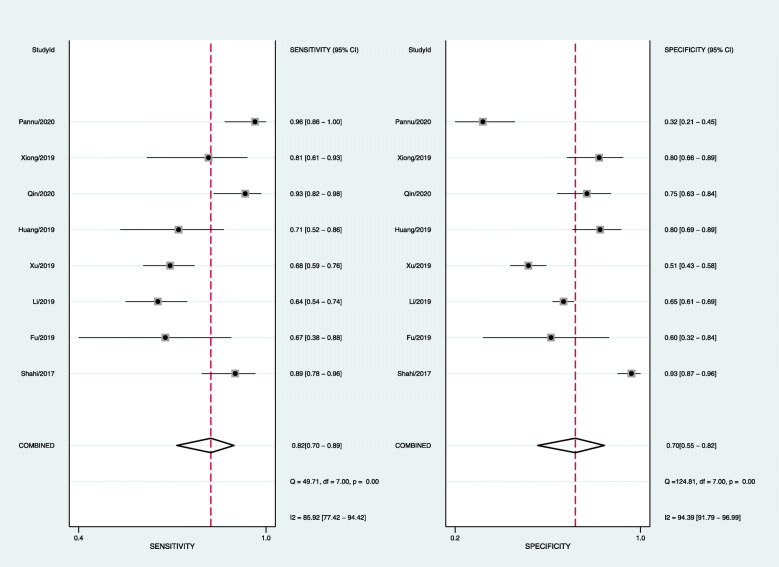
Forest plots of sensitivity and specificity

**Fig. 4:**
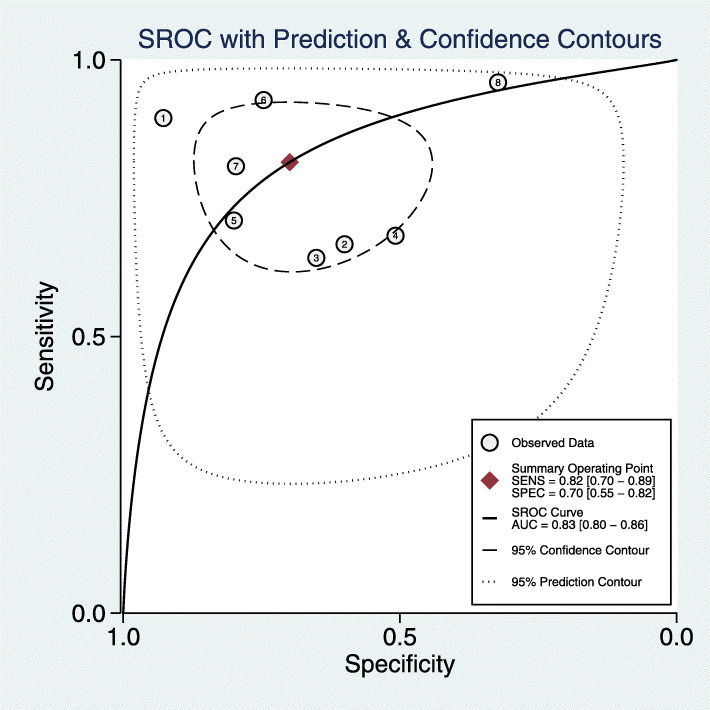
SROC curve of sequencing-based diagnosis performance

The pooled diagnostic sensitivity and specificity were 0.82 (95% CI, 0.70–0.89) and 0.70 (95% CI, 0.55–0.82), respectively (Fig. 3); however, the heterogeneity between studies was obvious, with *I*^2^ values of 83.19% (95% CI, 71.75–94.64%) and 94.17% (95% CI, 91.23–97.11%). The pooled PLR, NLR, and DOR were 2.7 (95% CI, 1.7–5.4), 0.26 (95% CI, 0.15–0.46), and 10 (95% CI, 4–25), respectively (Fig. 3). The AUC was 0.83 (95% CI, 0.8–0.86) (Fig. 4). The Spearman correlation coefficient was − 0.071 (*P* = 0.867). The heterogeneity might be unrelated to the threshold effects.

### Heterogeneity analysis

#### Meta-regression

**Fig. 5:**
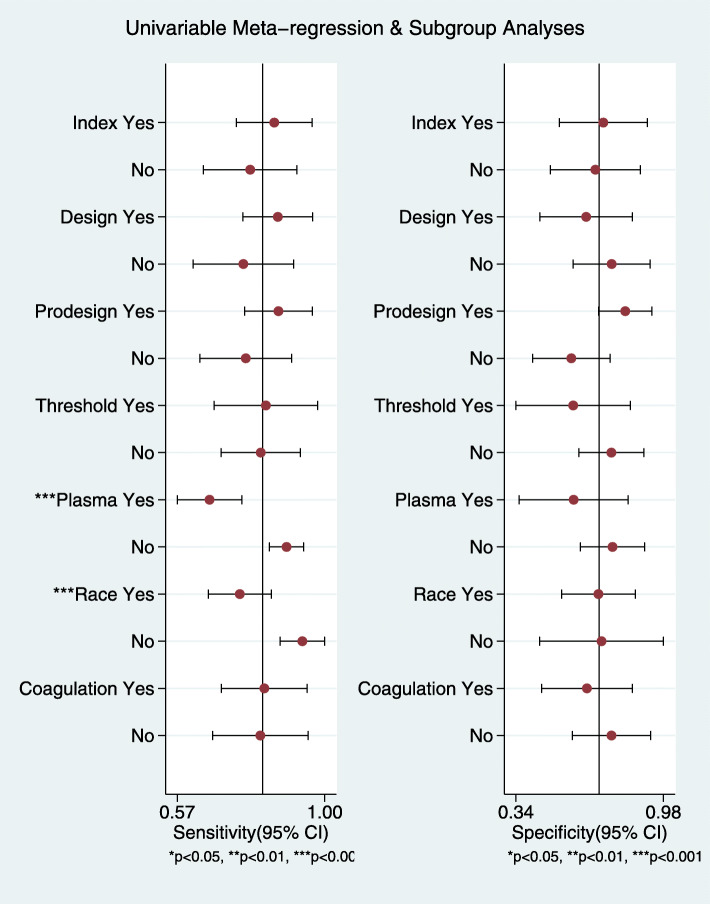
Univariable meta-regression

We performed univariate meta-regression to search for the potential sources of heterogeneity (Fig. 5). For sensitivity and specificity, the sample differences and racial differences had the most significant impacts on the heterogeneity of the results (*P* < 0.05). Based on these results, we performed subgroup analysis to further explore the source of heterogeneity. When *I*^2^ < 50% or *P* > 0.05, we considered the heterogeneity to be low in the subgroup.

### Subgroup analysis

In the subgroup of plasma D-dimer [18, 19, 22], the pooled sensitivity and specificity were 0.67 (95% CI 0.60–0.72) and 0.61 (95% CI 0.57–0.65); in the subgroup of serum D-dimer [16, 17, 20, 21, 23], the pooled sensitivity and specificity were 0.88 (95% CI 0.83–0.92) and 0.76 (95% CI 0.71–0.80). In the subgroup of East Asian races [16–19, 21, 22], the pooled sensitivity and specificity were 0.72 (95% CI 0.67–0.77) and 0.65 (95% CI 0.61–0.68); in the subgroup of Caucasian and African American races [20, 23], the pooled sensitivity and specificity were 0.92 (95% CI 0.86–0.97) and 0.74 (95% CI 0.67–0.80), respectively (Table 5).

**Table 5:** Subgroup analysis

Subgroup	Number of studies	Pooled sensitivity (95% CI)	Pooled specificity (95% CI)	***P***	***I***^**2**^
A	3	0.67(0.60–0.72)	0.61(0.57–0.65)	0.82/0.003	0/82.6%
B	5	0.88(0.83–0.92)	0.76(0.71–0.80)	0.0001/0.0001	68.7%/95.1%
C	6	0.72(0.67–0.77)	0.65(0.61–0.68)	0.001/0.0001	74.9%/84.5%
D	2	0.92(0.86–0.97)	0.74(0.67–0.80)	0.20/0.0001	39.5%/98.7%

*A* plasma D-dimer, *B* serum D-dimer, *C* East Asian race, *D* Caucasian and African American race

### Publication bias

**Fig. 6:**
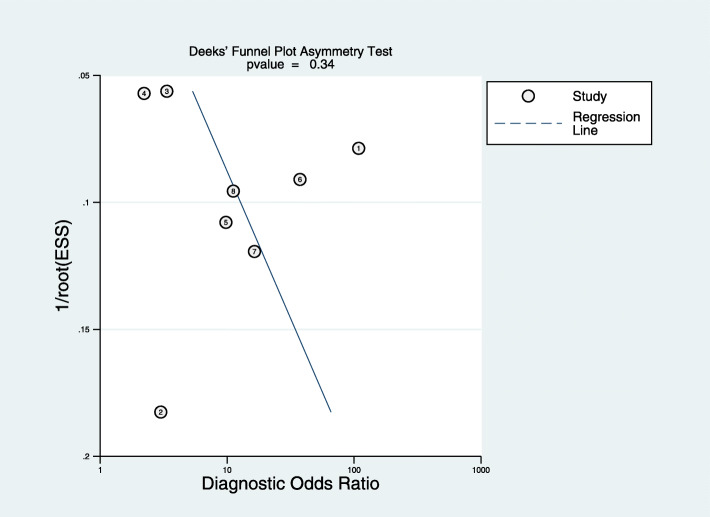
The Deeks’ funnel plot of the pooled DOR

The Deeks’ funnel plot asymmetry test of DOR did not show significant asymmetry (*P* = 0.34), indicating that publication bias might not exist (Fig. 6).

## Discussion

The diagnosis of PJI after arthroplasty is a complicated problem for every orthopedist. With early diagnosis, patients can undergo debridement or conservative treatment to treat PJI and avoid 1 or 2 stage revision. Therefore, the quick and accurate diagnosis of PJI is critical. Many potential blood and synovial fluid biomarkers for the diagnosis of PJI have been evaluated, but the clinical gold standard for the diagnosis of the disease has still not been found. Therefore, it is necessary and meaningful to develop a new and accurate diagnostic method for PJI.

D-dimer is familiar to medical workers and has not been valued in the past few decades. It has only been used to screen venous thromboembolism [24, 25]. Recently, some studies showed that D-dimer was associated with inflammation and might be elevated in infected patients [26, 27]. Rodelo et al. found that higher levels of D-dimer were associated with increased 28-day mortality in septic patients [9]. In addition, D-dimer is recommended as a critical diagnostic indicator for infectious diseases such as endocarditis and mycoplasma pneumonia [28, 29]. Subsequently, D-dimer levels attracted the attention of plastic surgeons.

Shahi et al. [20] reported in his study that serum D-dimer has high diagnostic value for PJI in lower limbs, with a sensitivity and specificity of 89% and 93%, respectively, which preluded the diagnosis of PJI by D-dimer. Parvizi et al. [30] believe that the diagnosis of PJI, such as ankylosing spondylitis, rheumatoid arthritis, and endocarditis, should depend on a combination of various diagnoses, so they added D-dimer and redefined the diagnosis of the PJI standard. The new diagnostic criteria were validated in 222 PJI patients and 200 sterile patients. They found that the sensitivity and specificity of the new diagnostic criteria were 97.7% and 99.5%, respectively, while the sensitivities of the MSIS and ICM diagnostic criteria were only 86.9% and 79.3%, and their specificities were both 99.5%. The ICM passed this diagnostic criterion in 2018, but the pass rate was only 68%. Since 2019, an increasing number of articles about D-dimer in the diagnosis of PJI have been reported, and its diagnostic value is suspected.

This is the first systematic review and meta-analysis about the utility of D-dimer for the diagnosis of PJI. We found that D-dimer has limited performance for the diagnosis of PJI, with a pooled sensitivity and specificity of 0.82 and 0.70, respectively, and had a poorer diagnostic value than that of CPR and ESR reported by Carli AV et al. [31]. In this systematic review, the pooled sensitivity and specificity of CRP were 0.85 and 0.81, respectively, and the pooled sensitivity and specificity of ESR were 0.82 and 0.79. The results of the subgroup analysis showed that serum D-dimer might have a higher diagnostic accuracy than plasma D-dimer for PJI (the pooled sensitivity was 0.88 vs 0.67, and the pooled specificity was 0.76 vs 0.61), and D-dimer had better accuracy in subgroups with Caucasian and African American races than in subgroups with East Asian races (the pooled sensitivity was 0.92 vs 0.72, and the pooled specificity was 0.74 vs 0.65).

One possible reason for the variance in the subgroup results was that the samples for the quantification of D-dimer were different: serum vs plasma. Serum is the liquid part of blood after coagulation, while plasma is the liquid part of the blood where coagulation has been prevented. Their density is similar, but their composition is different. The main difference is that there are more fibrinogen and coagulation proteins in plasma [32]. Boisclair et al. [33] reported that there was a very high correlation between plasma and serum D-dimer levels (*r* = 0.931, *P* < 0.01), but the diagnostic sensitivity was not consistent. The study reported that the sensitivities of plasma D-dimer for DIC, DVT, and MI were 100%, 90.4%, and 60%, respectively, but the sensitivities of serum D-dimer were 100%, 94.1%, and 22.2%. The different sensitivities of plasma and serum might be due to the more significant uncertainty in assigning a cut-off for elevated levels of serum D-dimer. The D-dimer assay was operating at its lower detection limit when used to measure non-elevated levels in serum [33]. However, whether different sensitivities between plasma and serum exist in PJI is not supported by relevant literature.

Another possible reason was that the level of D-dimer is easily affected by other diseases. Busso et al. [34] reported that the inflammatory synovium secretes a large amount of fibrin in patients with rheumatoid arthritis, and the degradation of this protein subsequently leads to an increase in the level of D-dimer in serum and synovial fluid. In addition, thrombosis [35], malignancies, autoimmune diseases, pregnancy, and heart and brain vascular diseases might affect the determination of D-dimer levels in the blood [36, 37]. Li et al. [19] found that the diagnostic accuracy of D-dimer was poor in the subgroups containing these diseases in their study.

In addition, racial differences may affect the diagnostic accuracy of D-dimer for PJI. Shahi and Pannu’s studies [20, 23] were conducted in the USA, and the population may be predominantly Caucasian and African-American. In the six other studies reported by Chinese scholars, the patients were predominantly of the East Asian race. The studies found that D-dimer levels varied between races, such as between African American and Caucasian patients [38, 39]. We suspect that there are also differences in D-dimer levels between the East Asian population and the other races, which will affect the result. However, there are no studies to support this view.

Synovial fluid viscosity tests and several other plasma biomarkers have been reported to diagnose PJI. The synovial fluid viscosity level was significantly lower in patients with PJI than in patients with aseptic failure, with a sensitivity of 0.99 and a specificity of 0.67 [22]. Both plasma fibrinogen and fibrin degradation product (FDP) are coagulation-related indicators. When the threshold for plasma fibrinogen was 4.01 g/L, the sensitivity and specificity values were 0.763 and 0.862 [19], respectively. FDP has low sensitivity and specificity, with values of 65.12% and 60.33%, respectively [18].

Our meta-analysis has some strengths and potential limitations. The cases included all involved hip and knee joints. In addition, all studies used MSIS standards [5] or modified MSIS standards [6]. Therefore, the classification bias was minimized. The most important factor was that all D-dimer tests were taken before surgery, excluding the interference of a sharp increase in serum D-dimer levels after surgery [40].

The limitations of our meta-analysis included variability in race, age range, sex ratio, and sample size. In addition, none of the studies considered whether patients used antibiotics before admission. Shahi et al. [20] reported that premature antibiotic treatment could affect the results of D-dimer in the blood. Another limitation of our study is that MSIS standards or modified MSIS standards lack the sensitivity to detect chronic and low-grade PJI; patients with “positive” D-dimer results might be classified as uninfected [41]. Additionally, most studies did not provide information about the measurement of D-dimer. D-dimer assays can be categorized into three types [42]: ELISA, immunoturbidimetric automated assay, and latex-based immunoassays. ELISA is more sensitive than immunoturbidimetric automated assays and latex-based immunoassays [42]. In addition, some studies excluded patients with tumors, rheumatoid arthritis, autoimmune diseases, a history of smoking, and obesity. However, the proportion of such patients in joint replacement is still high. The exclusion of these patients will interfere with the accuracy of D-dimer in the diagnosis of PJI. Finally, the diagnostic thresholds in some studies were not determined in advance, and the threshold values were not unified in this meta-analysis.

## Conclusion

D-dimer, a coagulation-related indicator, is inexpensive and easy to measure but has limited performance for the diagnosis of PJI, and the pooled sensitivity and specificity were poorer than those of traditional inflammatory markers such as CRP and ESR. Based on our findings, we suggest using serum samples for the quantification of D-dimer. Additionally, the diagnostic accuracy may be better in Caucasian and African American patients.

## Data Availability

Not applicable

## Abbreviations

PJI: Periprosthetic joint infection
PLR: Positive likelihood ratio
NLR: Negative likelihood ratio
DOR: Diagnostic odds ratio
AUC: The area under the SROC curve
MSIS: Musculoskeletal Infection Society
ICM: International Consensus Meeting
CRP: C-reactive protein
ESR: Erythrocyte sedimentation rate
WBC: White blood cell
PMN%: Polymorphonuclear neutrophil percentage
PRISMA: Preferred Reporting Items for Systematic Reviews and Meta-Analyses
